# Chitin deacetylases Cod4 and Cod7 are involved in polar growth of *Aspergillus fumigatus*

**DOI:** 10.1002/mbo3.943

**Published:** 2019-10-11

**Authors:** Mingming Xie, Xiaobing Zhao, Yang Lü, Cheng Jin

**Affiliations:** ^1^ State Key Laboratory of Mycology Institute of Microbiology Chinese Academy of Sciences Beijing China; ^2^ University of Chinese Academy of Sciences Beijing China; ^3^ Henan Agricultural University Zhengzhou China; ^4^ National Engineering Research Center for Non‐food Bio‐refinery Guangxi Academy of Sciences Nanning China

**Keywords:** *Aspergillus fumigatus*, cell wall, chitin, chitin deacetylase (CDA), polarity

## Abstract

Chitin is one of the key components of fungal cell wall, and chitin deacetylases (CDAs) have been found in fungi; however, their functions remain unknown. *Aspergillus fumigatus* is known to cause fatal invasive aspergillosis (IA) among immunocompromised patients with a high mortality rate. Although the *A. fumigatus* cell wall has long been taken as a unique target for drug development, its dynamic remodeling is complicated and not well understood. Seven putative CDAs are annotated in the *A. fumigatus* genome. In this study, we analyzed the function of the putative CDAs, Cod4 and Cod7, in *A. fumigatus*. Biochemical analysis of recombinant proteins showed that Cod4 preferentially deacetylated (GlcNAc)_4_ and was less active on chitooligosaccharides with DP > 5, whereas Cod7 was unable to catalyze deacetylation. Simulation of three‐dimensional structure revealed that both Cod4 and Cod7 shared a similar folding pattern with *Hy*PgdA from *Helicobacter pylori* and, similar to *Hy*PgdA, a substitution of Thr8 by Ala8 in Cod7 abolished its CDA activity. Deletion of the *cod4*, *cod7*, or both in *A. fumigatus* led to polarity abnormality and increased conidiation. Furthermore, the expression level of the genes related to polarity was upregulated in the mutants. Our results demonstrated that Cod4 and Cod7 were involved in polarity, though Cod4 was inactive.

## Graphical abstract

In our study, we show that *Aspergillus fumigatus* chitin deacetylase Cod4 preferentially deacetylated tetra‐*N*‐acetylglucosamine, (GlcNAc)_4_, whereas Cod7 is inactive, possibly due to a substitution of threonine by alanine at position 8. Deletion of cod4, cod7, or both leads to abnormal polarity and increased conidiation. Our results demonstrate that both Cod4 and Cod7 are involved in polarity.

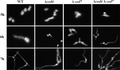

## 1 INTRODUCTION

*Aspergillus fumigatus*, a saprophytic filamentous fungus, is known to cause fatal invasive aspergillosis (IA) among immunocompromised patients, and in recent years, the incidence of IA has soared up in patients who undergo immunosuppressive and cytotoxic chemotherapy (Latgé, 1999; Steinbach, Stevens, & Denning, 2003). Although three classes of antifungal drugs, including antifungal triazoles, amphotericin B, and echinocandin, have been currently used (Walsh et al., 2008), the mortality rate remains around 50% due to limited drug options, low efficiency, and drug resistance (Brown et al., 2012; Denning & Bromley, 2015). Therefore, new antifungal therapies are urgently needed.

The *A. fumigatus* cell wall is essential for survival, and its components are critical for fungal pathogenesis. Therefore, it is a unique target for drug development. Generally, a filamentous fungus initiates its life cycle from conidial germination, continues with hyphal growth, and terminates with conidiation, which involves a series of ordered morphological events, including the establishment of polarity (budding and germination), polar growth (mycelia elongation, septation, and branching), and conidiation (Barhoom & Sharon, 2004; d'Enfert, 1997). These morphological events require a dynamic remodeling of the cell wall at the budding site, hyphal tip, and septation site. IA caused by *A. fumigatus* is featured with the penetration of growing hyphae into lung tissue and blood vessel. Therefore, the polar growth of *A. fumigatus* is vital for infection. However, it is still not well understood how *A. fumigatus* modulates the remodeling of cell wall during polar growth.

Chitin, an unbranched polymer of N‐acetylglucosamine (GlcNAc), is a major component of the cells of most filamentous fungi (Bartnicki‐Garcia, 1968) and contributes to the strength and integrity of the fungal cell wall and septum (Minke & Blackwell, 1978). It has been known that the cell wall chitin is predominantly deacetylated to chitosan, which is enzymatically generated by chitin deacetylase (CDA). CDAs have been identified in bacteria (Kadokura et al., 2007; Li, Wang, Wang, & Roseman, 2007), insects (Dixit et al., 2008), and fungi (reviewed in Zhao, Park, & Muzzarelli, 2010). According to the CAZy classification, CDAs (EC3.5.1.41) belong to the family 4 of carbohydrate esterases (CE4) and share a conserved part of the primary amino acid structure named as the NodB homology domain or polysaccharide deacetylase domain (Caufrier, Martinou, Dupont, & Bouriotis, 2003; Lombard, Golaconda Ramulu, Drula, Coutinho, & Henrissat, 2014). Putative members of the CE4 family are abundant in the genomes of chitin‐containing fungi (http://www.cazy.org).

In fungi, CDAs participate in cell wall morphogenesis and integrity, spore formation, germling adhesion, and fungal autolysis (Baker, Specht, Donlin, & Lodge, 2007; Davis & Bartnicki‐Garcia, 1984; Geoghegan & Gurr, 2016; Matsuo, Tanaka, Matsuda, & Kawamukai, 2005; Zhao et al., 2010). Deacetylation of chitin in the cell wall affects virulence of*Cryptococcus neoformans* (Baker et al., 2007; El Gueddari, Rauchhaus, Moerschbacher, & Deising, 2002), *Colletotrichum lindemuthianum* (Blair et al., 2006), *Magnaporthe oryzae* (Kuroki et al., 2017), *Pochonia chlamydosporia* (Aranda‐Martinez et al., 2018), and *Pestalotiopsis*sp. (Cord‐Landwehr, Melcher, Kolkenbrock, & Moerschbacher, 2016). CDAs have been biochemically characterized with regard to substrate specificity in several fungi, such as *Mucor rouxii* (Kafetzopoulos, Martinou, & Bouriotis, 1993), *Aspergillus nidulans* (Alfonso, Nuero, Santamaría, & Reyes, 1995; Liu et al., 2017), *C. lindemuthianum* (Blair et al., 2006; Tsigos & Bouriotis, 1995), *Puccinia graminis* (Naqvi et al., 2016), *Pestalotiopsis*sp. (Cord‐Landwehr et al., 2016), and *Podospora anserina* (Hoßbach et al., 2018). They preferentially deacetylate (GlcNAc)_4_ and (GlcNAc)_5_ with random, sequential, or processive mechanisms.

Chitin is one of the critical components of the *A. fumigatus* cell wall (Bernard & Latgé, 2001), and the genome of *A. fumigatus* contains seven putative CDA genes; however, their functions remain unknown. In this study, we cloned the *A. fumigatus cod4* and *cod7* genes and expressed them in *E. coli* with an appended short N‐terminal His‐tag. Biochemical characterization of the recombinant enzymes was carried out. We further deleted the *cod4* and *cod7* separately as well as both of them to construct the deletion mutants ∆*cod4*, ∆*cod7*, and ∆*cod4*∆*cod7*. Our results showed that the Cod4 was an active CDA, whereas Cod7 was inactive; however, both of them contributed to polar growth and Cod4 was required for conidiation of *A. fumigatus*.

## 2 EXPERIMENTAL PROCEDURES

### 2.1 Strains and growth conditions

*Aspergillus fumigatus* strain YJ‐407 (China General Microbiological Culture Collection Center, CGMCC0386) was maintained on potato glucose (2%) agar slant. *A. fumigatus* strain CEA17 (Weidner, d'Enfert, Koch, Mol, & Brakhage, 1998), a kind gift from C. d'Enfert, Institute of Pasteur, France, was propagated at 37°C on YGA (0.5% yeast extract, 2% glucose, and 1.5% Bacto agar), complete medium, or minimal medium with 0.5 mM sodium glutamate as a nitrogen source (Cove, 1966). 5 mM uridine and uracil were added when CEA17 or revertant strain was grown. Mycelium was grown in complete liquid medium at 37°C with constant shaking at 250 rpm. Mycelia were collected and washed with distilled water, and then frozen in liquid nitrogen and ground by pestles. The powder was then stored at −70°C for DNA, RNA, and protein extraction. Conidiospores were acquired by growing *A. fumigatus* strains on PDA plates with uridine and uracil for 36 hr at 37°C. The spores were collected first with distilled water, then washed twice with 0.05% Tween‐20 in phosphate‐buffered saline (PBS), and stored in 0.05% Tween‐20 in PBS, and its concentration was confirmed via hemocytometer counting. Vectors and plasmids were propagated in *Escherichia coli* Trans‐T1 (TransGen Biotech).

### 2.2 Molecular cloning of the cod4 and cod7

Protein sequences were analyzed using Conserved Domain Search, SignalP3.0, and the TMHMM Server v.2.0. Protein sequence and AFUA number were retrieved from the CADRE Geno. Protein sequences were aligned using ClustalX 2.0.

The putative CDA gene was identified by searching the conserved domain of NodB that is homologous among the members of carbohydrate esterase family 4 in the genome database of *A. fumigatus* 293 using a Blastp program. A 1,040‐bp and a 915‐bp genomic DNA fragment were found to contain the entire ORF and named as *cod4* and *cod7*, respectively.

Based on the nucleotide sequence, the primer pair cod4p1 (5′‐ATGGGCAAGAAGCGCGT TCT‐3′)/cod4p2 (5′‐TTATTTCTTGAGAATGGCCC‐3′) and cod7p1 (5′‐ATGGGCAAG AAACGTGTATT‐3′)/cod7p2 (5′‐CTCCAAAACCGGAGTAACCG‐3′) were used to clone the cDNA of the *cod4* and *cod7*genes by PCR, respectively. The PCR products were subcloned into the Trans‐T‐Easy vector (TransGene) to obtain Teasy‐cod4 and Teasy‐cod7.

### 2.3 Expression and purification of recombinant Cod4 and Cod7 in *E. coli*

The cDNA of the *A. fumigatus cod4* or *cod7* gene was amplified from Teasy‐cod4 or Teasy‐cod4 and subcloned into pET‐21a (Novagen). The recombinant *E. coli *BL21(DE3) strain (Novagen) harboring pET21‐cod4 or pET21‐cod7 was used to express the recombinant protein Cod4 or Cod7. One percent of transformant was inoculated into LB containing 100 µg/ml ampicillin and 50 µg/ml chloramphenicol, incubated at 37°C and 200 rpm for 2–3 hr to reach 0.6 at OD_600_, and then induced with a final concentration of 0.4 mM of isopropyl‐*β*‐d‐thiogalactopyranoside (IPTG) at 28°C for 4 hr. The recombinant protein was purified by ÄKTA FPLC (GE Pharmacia) with a HisTrap HP column (Amersham Pharmacia Biotech). After purification, proteins were dialyzed, lyophilized, and stored at −70°C for later use. The purity of Cod4 or Cod7 was judged by SDS‐PAGE, and the concentration was determined by the BCA method.

### 2.4 Activity assay

The assay of CDA was carried out by the acetic acid released from the substrates (Fukushima, Kitajima, & Sekiguchi, 2005). Standard enzyme assay was performed in a mixture containing 20 mM Tris‐HCl (pH 7.4), 1 mM ZnCl_2_, and 5 μl of pNP‐(GlcNAc)_3_ or (GlcNAc)_4_ (1 mg/ml) in a total volume of 100 μl. The reaction was carried out at 37°C for 30 min and then stopped in boiled water for 10 min. Acetic acid released by the enzyme was quantified with the K‐ACET kit (Megazyme). The amount of enzyme that releases 1 μmol of acetate from ethylene glycol chitin per minute is defined as one unit.

### 2.5 Construction of the mutants and revertants

To delete the *cod4*, a knockout vector construction was designed to replace the entire coding region of the *cod4* with a *pyrG* cassette by homologous recombination (d'Enfert et al., 1996). PCR primers were designed to amplify a 1.8‐kb upstream noncoding region (5′‐GGTGGTGCGGCCGTTCTGACTGCCCGATAT‐3′ and 5′‐GGTGGTTCTAGAGAC CCGATCTTGGCGCTCTTT‐3′; underlines indicate introduced *Not*I or *Xba*I site) of the *cod4* before the ATG start codon and a 1.8‐kb downstream noncoding region (5′‐GGTGGTTCTAGATTTGTGAAGAATCGGCCTACT‐3′ and 5′‐GGTGGTGGTACCACAGTGGCGGCCG CGGCGAGTTT‐3′; introduced *Xba*I and *Kpn*I sites are underlined) of the *cod4* after the stop codon. The upstream and downstream noncoding regions were digested with *Not*I/*Xba*I and *Xba*I/*Kpn*I, respectively, and then cloned into the relevant sites of Trans‐T‐Easy linear vector (TransGen Biotech). The *pyrG* blaster cassette (8.6 kb) released by the digestion of pCDA14 (d'Enfert et al., 1996) with *Hpa*I was cloned into the site between the up‐ and downstream noncoding regions of the *cod4* to yield the deletion construct pcod4‐pyrG. At a unique *Not*I site, the linearized pcod4‐pyrG was transformed into strain CEA17 by protoplast transformation (Yelton, Hamer, & Timberlake, 1984) and screened for transformants with uridine and uracil autotrophy. The PCR confirmation of the mutant was done by using primer pairs of 5′‐CTAGAGGTAAGTAATCAGTAAC‐3′/5′‐CTTCCTAATACCGCCTAGTC‐3′ for the *pyrG* and 5′‐ATGGGCAAGAAGCGCGTT CT‐3′/5′‐TTTCTTGAGAATGGCCCCCG‐3′ for the *cod4*. Southern blot was carried out by using a 1‐Kb fragment as a probe, which was amplified from the upstream noncoding region of the *cod4* gene using primers 5′‐GGACCCCAGCGACTGCAATG‐3′ and 5′‐TTCTGCAATTTTGAGCTCGT‐3′.

To construct deletion mutant of the *cod7*, primers were designed to generated the upstream noncoding region (5′‐GGTGCGGCCGCCAATATCAAAAAAGTCGATACGACTT‐3′ and 5′‐GGTCTCGAGGATCCCGAATTGAGTACTGAG‐3′; introduced *Not*I and *Xho*I sites are underlined) and downstream noncoding region (5′‐GGTCTCGAGAGCTGGT ATCCGGCCGAGCT‐3′ and 5′‐GGTGGTACCCCTAGCAGGGCCGATGTGCTG‐3′; introduced *Xho*I and *Kpn*I sites are underlined) of the *cod7*. The mutant Δ*cod7* was obtained by knocking out the *cod7* gene in the wild type with a similar protocol as described above. PCR confirmation of the Δ*cod7* mutant was carried out using primer pairs of 5′‐CTAGAGGTAAGTAATCAGTAAC‐3′/5′‐CTTCCTAATACCGCCTAGTC‐3′ to amplify the *pyrG* and 5′‐GGTTCAGCTGCTGCCTGAGGCTGGACG‐3′/5′‐AGGAAGG CTTTGTGTTTGATC‐3′ to amplify the *neo*. A 1‐Kb fragment was amplified from the upstream noncoding region of the *cod7* gene using the primers 5′‐GTCGCAGTCAGGG AAGCAAG‐3′ and 5′‐ATCCCGAATTGAGTACTGA‐3′ and used as a probe to confirm the Δ*cod7* mutant.

To generate the revertant strains or double mutant, the *pyrG* gene was first deleted in the Δ*cod4* or Δ*cod7* mutant using the method described by d'Enfert et al. (1996) to generate the Δ*cod4*Δ*pyrG* or Δ*cod7*Δ*pyrG* strain. Then, the wild‐type copy of the *cod4* or *cod7* was transformed into the Δ*cod4*Δ*pyrG* or Δ*cod7*Δ*pyrG* strain to replace *pyrG*, respectively. The double mutant was constructed by deletion of the *cod7* gene in the Δ*cod4*Δ*pyrG* strain.

### 2.6 Phenotypic analyses of the mutants

Growth kinetics of *A. fumigatus* strains was carried out by spotting 1 × 10^6^ conidiospores onto the center of a solid CM plate at 37°C or 50°C, respectively. The diameter of the colony was measured intermittently until the stationary phase, and the mean diameter was used to plot against the growth kinetics. This experiment was carried out in triplicate.

To test the sensitivity to antifungal reagents, the same amount of conidiospores freshly collected from the wild type, the mutants, and the revertant strains was spotted on CMU plates in the presence of 250 μg/ml calcofluor white or 250 μg/ml Congo red. After incubation at 37°C or 50°C for 24–48 hr, the plates were taken out and photographed.

To examine spore germination, 10 ml complete liquid medium was inoculated with 10^7^ freshly harvested conidia, poured into a petri dish containing a glass coverslip, and incubated at 37°C for the time indicated in each experiment. At the specified times, the coverslides with adhering germlings were removed and fixed in a fixative solution (4% formaldehyde, 50 mM phosphate buffer, pH 7.0, and 0.2% Triton X‐100) for 30 min. The coverslips with geminated spores were then washed with phosphate‐buffered saline (PBS), incubated for 15 min with DAPI (1 μg/ml; Sigma), washed with PBS three times, then incubated for 5 min with a 10 μg/ml solution of fluorescent brightener 28 (Sigma), and washed again, and the germlings were photographed under microscope.

To count the number of germ tubes during spore germination, 10 ml of liquid CM was inoculated with 10^6^ spores in a petri plate containing sterilized glass coverslips and incubated at 37°C or 50°C. The coverslips with adhering germinated conidia were taken out, fixed in PFA solution (3.7% paraformaldehyde, 50 mM phosphate buffer, pH 7.0, and 0.1% Triton X‐100), and observed and counted under differential interference contrast microscope.

Conidia were fixed in 2.5% glutaraldehyde in 0.1 M phosphate buffer (pH 7.0) for 4 hr or overnight at 4°C. Cells were fixed in 2.5% glutaraldehyde in 0.1 M phosphate, washed three times in 0.1 M phosphate, postfixed in 1% osmium tetroxide, for 2–4 hr in 0.1 M phosphate and then for 15–20 min in methanol 30%, 50%, 70%, 85%, 95%, and 100%, respectively, and postfixed in 2% uranyl acetate–methanol 30%. Cells were rinsed, dehydrated, and embedded in Epon 812 for the floating sheet method. The section was examined with H‐600 electron microscope (Hitachi).

### 2.7 Analysis of the cell wall

Cell wall components were isolated and determined as described by Yan et al. (2013). Cell wall chitin was isolated as described by White, Farina, and Fulton (1979). The purification procedures included an alkaline treatment, which followed an acidic environment. Deacetylation degree of chitin was determined by the IR spectrophotometry method (Muzzarelli, Tanfani, Scarpini, & Laterza, 1980).

### 2.8 Real‐time PCR

Examination of the expression level of genes by relative real‐time RT‐PCR analysis was performed as described previously (Yan et al., 2013). The primers used for specific genes in this study are shown in Table 1. First, total RNAs were extracted using TRIzol (Invitrogen). The cDNA synthesis was carried out from total RNA using the RevertAid™ First Strand cDNA Synthesis Kit (Fermentas). Then, the PCR was performed by using SYBR^®^ *Premix Ex Taq*™ (Takara). A triplicate of samples was tested in each assay, and each experiment was repeated 3 times. To testify the contamination of fungal genomic DNA, negative controls were set up for each gene.

**Table 1:** Primers used in polarity and virulence analyses by real‐time PCR

	Forward primer	Reverse primer
TBP	5′‐CCACCTTGCAAAACATTGTT‐3′	5′‐TACTCTGCATTTCGCGCATG‐3′
*cdc42*	5′‐GGAGCTGGGTGCTGTAAAATACGTC‐3′	5′‐GCCGCAACAATCGCCTCATC‐3′
*rsr1*	5′‐GTTGCCTTACTGCTCAATTCGTT‐3′	5′‐TCTGCTTTCGATAGGAGTCTTCAAT‐3′
*rho3*	5′‐ATACCTTGAATGCTCTGCTG‐3′	5′‐TTCACATCAAGAGCGACCTT‐3′
*rho1*	5′‐TTTCATACCCCGACTCCCACG‐3′	5′‐CGGAGATCCACTTCTCCTGG‐3′
*sur2*	5′‐GGCTTTACGTCACTTTCCACTCTCG‐3′	5′‐AAGCCTTCCACCGGGTGATT‐3′
*sepA*	5′‐TGGTGGAGTGGAAGAAATCGAAAG‐3′	5′‐GCCGGCGTTGACACGTTTGC‐3′
*kipA*	5′‐CTATTTATATGACAATGTCTTCCCGC‐3′	5′‐CTTCTTACAAGGCGCTTGGC‐3′
*lag1*	5′‐GTTGAGTTACCGGTTCCACT ‐3′	5′‐TTTTTGAGGTCGCTAGGAAG‐3′
*swoC*	5′‐AACTATGACAAGCTCATGGTGAGG‐3′	5′‐TTCAGGTCACGCTTGAGAGG‐3′
*amb*	5′‐GAATGACTGTGGACGATGATGTG‐3′	5′‐GGATGTCGATGGCCAGTTG‐3′
*pksP*	5′‐TGGTGGAGTGGAAGAAATCGAAAG‐3′	5′‐GCCGGCGTTGACACGTTTGC‐3′
*fos‐1*	5′‐CTATTTATATGACAATGTCTTCCCGC‐3′	5′‐CTTCTTACAAGGCGCTTGGC‐3′
*rhbA*	5′‐GTTGAGTTACCGGTTCCACT ‐3′	5′‐TTTTTGAGGTCGCTAGGAAG‐3′
*pabA*	5′‐AACTATGACAAGCTCATGGTGAGG‐3′	5′‐TTCAGGTCACGCTTGAGAGG‐3′
*lysF*	5′‐GAATGACTGTGGACGATGATGTG‐3′	5′‐GGATGTCGATGGCCAGTTG‐3′

## 3 RESULTS AND DISCUSSION

### 3.1 Expression and biochemical characterization of recombinant CDAs

Based on the Blast results, seven genes were found to encode putative CDAs, named as *cod1‐7*. Analysis of the complete cDNA sequences with SignalP4.1 (http://www.cbs.dtu.dk/services/SignalP/) predicted that the *cod4* (AFUA_5G09130) and *cod7* (AFUA_6G05030) were soluble CDAs without signal peptide or transmembrane domain, suggesting these two CDAs are soluble proteins and different from other ones that have been reported. To understand the functions of these two soluble CDAs, both *cod4* and *cod7*genes were chosen for further study. The *cod4* gene is 1,040 bp in length and contains two introns and three exons. A 927‐bp region of the *cod4* cDNA encodes a protein of 308 aa (XP_753725.1). The *cod7* gene is 1,111 bp in length and contains three introns and four exons. A 918‐bp region of the *cod7* cDNA encodes a protein of 306 aa (XM_742482.1). Cod4 and Cod7 share a similarity of 80%.

**Figure 1:**
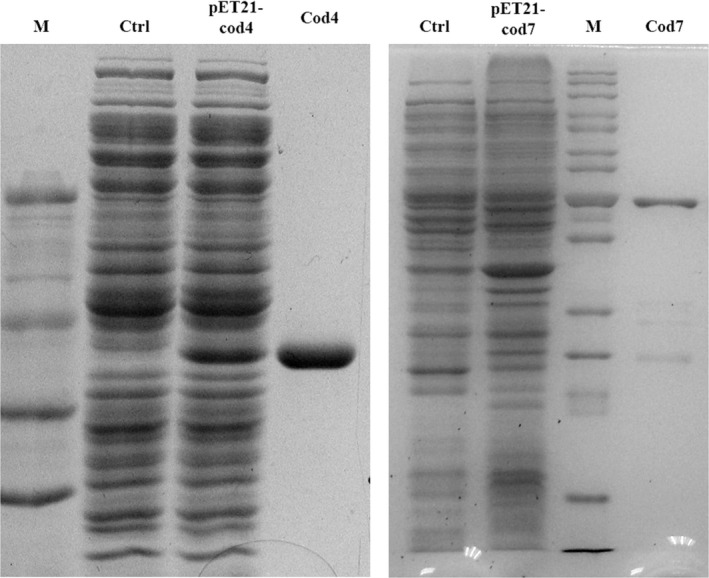
Expression and purification of Cod4 and Cod7. *E. coli* harboring pET21‐cod4 or pET21‐cod7 was induced with 0.6 mM IPTG at 28°C for 10 hr. Recombinant proteins were purified by ÄKTA FPLC system (GE Pharmacia) with a HisTrap HP column (Amersham Pharmacia Biotech). The purity of Cod4 or Cod7 was judged by 10% of SDS‐PAGE. Ctrl, *E. coli* harboring pET‐21a; M, marker

The cDNA of the *cod4* or *cod7* gene was cloned and expressed in *E. coli *BL21(DE3). The recombinant Cod4 and Cod7, containing a short N‐terminal His‐tag, were purified to homogeneity by Ni^2+^ affinity chromatography. A 35‐kDa of recombinant Cod4 and Cod7 protein were purified to homogeneity, respectively (Figure 1).

**Figure 2:**
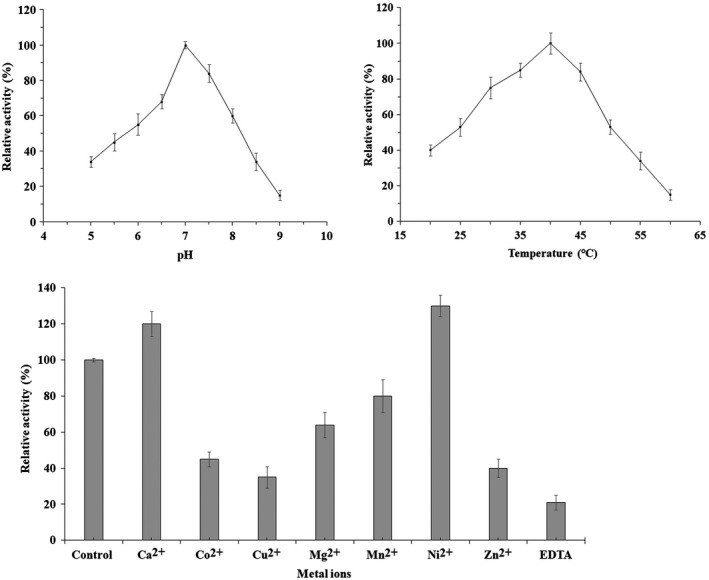
Effect of pH, temperature, and metal ions on activity of Cod4. Standard activity assay was carried out by adding 10 μg of purified Cod4 to a mixture containing 20 mM Tris‐HCl (pH 7.4), 1 mM ZnCl_2_, and 5 μl of 1 mg/ml pNP‐(GlcNAc_3_) in a total volume of 100 μl, and the reaction mixture was incubated at 37°C for 30 min. Reactions were terminated by boiling the mixture at 100°C for 10 min. Acetic acid released by the enzyme was quantified with the K‐ACET kit (Megazyme). The amount of enzyme that releases 1 μmol of acetate per minute is defined as one unit. To determine the temperature optimum for activity, reactions were performed at 20–60°C under otherwise standard conditions. To determine the pH optimum for activity, reactions were performed at pH 5.0–9.0 under otherwise standard conditions. The effect of metal ions was tested by adding 5 mM of EDTA or various metal ions to the reaction mixtures. Control, Zn^2+^

Under standard assay conditions, a variety of substrates were tested. As summarized in Table 2, Cod4 showed the highest CDA activity toward pNP‐β‐(GlcNAc)_3_ and (GlcNAc)_4_, deacetylated pNP‐β‐GlcNAc, (GlcNAc)_2_ and (GlcNAc)_5_ at a lower rate, and no activity toward pNP‐β‐(GlcNAc)_5‐6_, GlcNAc, and (GlcNAc)_6_. In addition, Cod4 was inactive on 4‐nitrophenyl butyrate, 4‐nitrophenyl palmitate, 4‐nitrophenyl, and acetylxylan. These results clearly demonstrate that Cod4 is an active CDA in *A. fumigatus*. Studies of the effect of temperature and pH on CDA activity of Cod4 toward pNP‐β‐(GlcNAc)_3_ showed optima of approximately 37°C and pH 7.1, respectively (Figure 2).

**Table 2:** Substrate specificity of Cod4

Substrate	Activity (U/mg)	Substrate	Activity (U/mg)
pNP‐GlcNAc	0.75	GlcNAc	1.47
pNP‐(GlcNAc)_2_	1.34	GlcNAc_2_	3.57
pNP‐(GlcNAc)_3_	2.58	GlcNAc_3_	13.45
pNP‐(GlcNAc)_4_	2.37	GlcNAc_4_	18.98
pNP‐(GlcNAc)_5_	Nd	GlcNAc_5_	4.56
pNP‐(GlcNAc)_6_	Nd	GlcNAc_6_	1.34

Note

Standard enzyme assays were performed in a mixture containing 20 mM Tris‐HCl (pH 7.4), 1 mM ZnCl_2_, and 5 μl of 1 mg/ml pNP‐(GlcNAc)_1–6_ or (GlcNAc)_1–6_. Reaction mixture was incubated at 37°C for 30 min and then boiled at 100°C for 10 min to stop the reaction. Acetic acid released by the enzyme was quantified with the K‐ACET kit (Megazyme). The amount of enzyme that releases 1 μmol of acetate from ethylene glycol chitin per minute is defined as one unit.

The activity of CE4 deacetylases depends on bivalent metals (Blair, Schuttelkopf, MacRae, & Aalten, 2005; Taylor et al., 2006). In our study, the CDA activity of Cod4 was completely inhibited by the addition of 5 mM EDTA. Of several bivalent metal ions tested, Zn^2+^, Ni^2+^, and Ca^2+^ were beneficial for activity, while Mn^2+^, Fe^2+^, Co^2+^, Cu^2+^, and Mg^2+^ inhibited the CDA activity (Figure 2). These results indicate that Cod4 is different from *An*CDA (Liu et al., 2017).

Several fungal CDAs have been found to fully deacetylate (GlcNAc)_4_ and (GlcNAc)_5_, whereas their activity toward the reducing‐end residue of chitooligosaccharides is undetectable (Zhao et al., 2010). For example, CDA from *C. lindemuthianum* can fully deacetylate (GlcNAc)_3_ and (GlcNAc)_4_, whereas the reducing‐end residue of (GlcNAc)_2_ could not be deacetylated (Tokuyasu, Ono, Ohnishi‐Kameyama, Hayashi, & Mori, 1997). It seems justified to conclude that the four fungal CDAs analyzed in detail so far all prefer to deacetylate the position next to the reducing end when chitin tetramer is used as a substrate, but they differ in the further conversions of the substrate (Hoßbach et al., 2018).

In this study by using pNP‐(GlcNAc)_1‐6_ as substrates, which are chitooligosaccharides labeled by p‐nitrophenol at their reducing end, we observed a low CDA activity toward pNP‐GlcNAc. Almost no activity toward GlcNAc was detected. These results demonstrate that Cod4 is inactive on the reducing end of chitooligosaccharides and similar to the CDAs from other fungi. On the other hand, Cod4 showed the highest activity toward pNP‐(GlcNAc)_3_and (GlcNAc)_4_. As p‐nitrophenol ring can be treated as a sugar ring analog at the reducing end, it is reasonable to conclude that Cod4 prefers (GlcNAc)_4_ and is less active on chitooligosaccharides with DP > 5.

**Figure 3:**
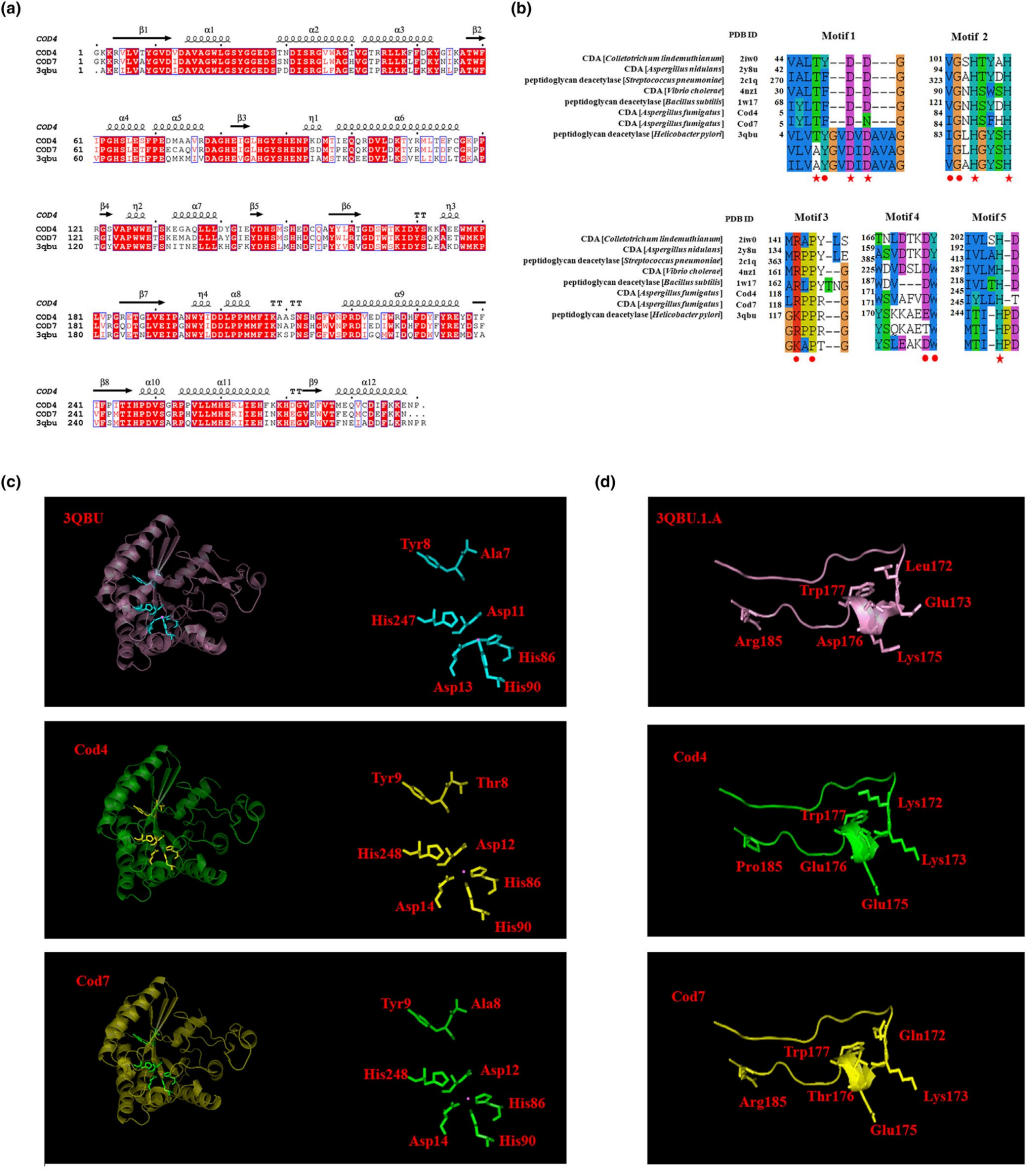
Comparison of Cod4 and Cod7 with other CE4 family members. (a) Comparison of the secondary structures of Cod4, Cod7, and *Hp*PgdA; (b) alignment of fungal CDAs and bacterial peptidoglycan deacetylases; (c) predicted three‐dimensional structures of Cod4 and Cod7 (SWISS‐MODEL at http://swissmodel.expasy.org/); and (d) predicted subunit binding domain. The red star represents the catalytic amino acid, the red dot represents metal‐binding amino acid, and the pink dot represents Zn^2+^

**Figure 4:**
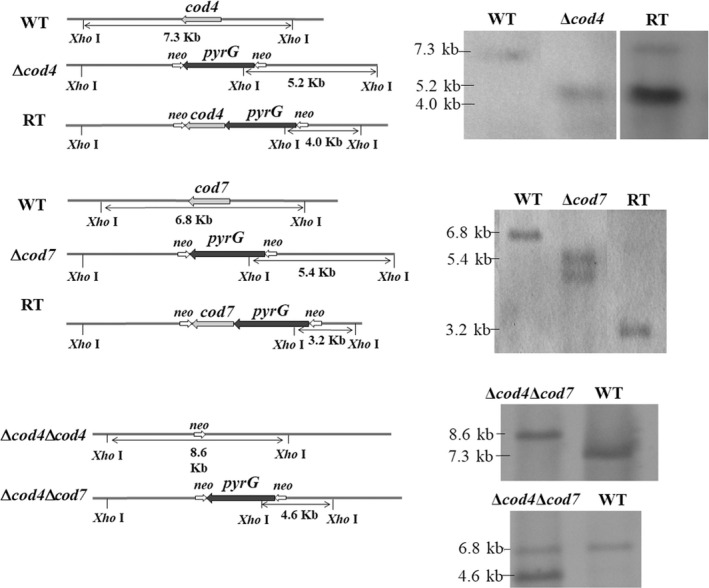
Southern blotting of the mutants and revertants. The mutants and revertant strains were constructed as described under Section 2. After PCR confirmation of the mutants and revertants, the positive strains were further confirmed by Southern blot using a 1‐kb fragment amplified from the upstream noncoding region of the *cod4* or cod7 gene as a probe

It is proposed that CDAs have four subsites (−2, −1, 0, and +1). The enzyme strongly recognizes a sequence of four GlcNAc residues of the substrate, and the *N*‐acetyl group in the GlcNAc residue positioned at subsite 0 is exclusively deacetylated. More recently, Liu et al. (2017) described the crystal structure and substrate‐binding modes of *An*CDA (XP_682649.1) from *A. nidulans*. *An*CDA is active toward (GlcNAc)_2–6_ and inactive toward the GlcNAc and mono‐deacetylation of (GlcNAc)_2_. It has been confirmed that deacetylation catalyzed by *An*CDA occurs at random positions, except for the reducing end. *An*CDA adopts (β/α)8 barrel topology. Similar to *Cl*CDA (Blair et al., 2006; Sarkar, Gupta, Chakraborty, Senapati, & Gachhui, 2017), the active site of *An*CDA contains the His‐His‐Asp metal‐binding triad (H97, H101, D48), a catalytic acid (His196, aiding sugar departure), and a catalytic base (Asp47, activating the nucleophilic water). The −1 sugar can make several interactions with Asp47 and His101, while the sugar bound in subsite +1 interacts with Leu139 and Leu194 that form a hydrophobic pocket, which is important for effective catalysis and preferable deacetylated site. Cod4 contains the His‐His‐Asp metal‐binding triad (H87, H91, D14), a catalytic acid His248 and a catalytic base Asp12 (Figure 4b). As compared with *An*CDA, it seems that Asp12 and His91 are responsible for interaction with the −1 sugar, while at subsite + 1 the counterparts of Leu139 and Leu194 were not found; indeed, they are replaced by hydrophobic amino acids Gly123 and Ile247, respectively (Figure 3b).

On the other hand, in contrast to Cod4, Cod7 did not show any CDA activity toward the substrates tested in this study though Cod7 shares a similarity of 80% with Cod4. We further compared these two proteins with other reported members of the CE4 family (Andrés et al., 2014; Blair & van Aalten, 2004; Blair et al., 2005; Fadouloglou et al., 2017; Shaik, Cendron, Percudani, & Zanotti, 2011), including alignment, secondary structure, and three‐dimensional structure. Blast search revealed that Cod4 and Cod7 shared 67% of similarity with a putative peptidoglycan deacetylase from *Helicobacter pylori* (*Hp*PgdA; Shaik et al., 2011). As shown in Figure 4a, Cod4 and Cod7 share a similar folding pattern with *Hp*PgdA (PDB ID: http://www.rcsb.org/pdb/search/structidSearch.do?structureId=3QBU). Therefore, three‐dimensional structures of Cod4 and Cod7 were simulated with SWISS‐MODEL (Figure 3c). As indicated in Figure 4b,c, the amino acid residues required for catalysis and Zn^2+^ binding are conserved in Cod4, Cod7, and *Hp*PgdA except the Motif 1, in which the conserved TF(/Y)DD are AYDD in both Cod7 and *Hp*PgdA (Figure 3b,c). Like other CDAs, in Cod4 a catalytic triad, T8‐D12‐H248, is identified to be required for deacetylation and H87‐H91‐D14 is for Zn^2+^ binding. In *Hp*PgdA, the corresponding residues of the catalytic triad Thr‐Asp‐His are A7, D11, and H247. Previously, it has been shown that *Hp*PgdA is inactive on peptidoglycans and polyamines. Similarly, Cod7 was inactive on N‐acetyl‐oligosaccharides. Therefore, it is reasonable to conclude that, as in *Hp*PgdA, Cod7 is unable to catalyze deacetylation due to the substitution of T^8^ by A^8^.

### 3.2 Functional analyses of the cod4 and cod7 gene

**Figure 5:**
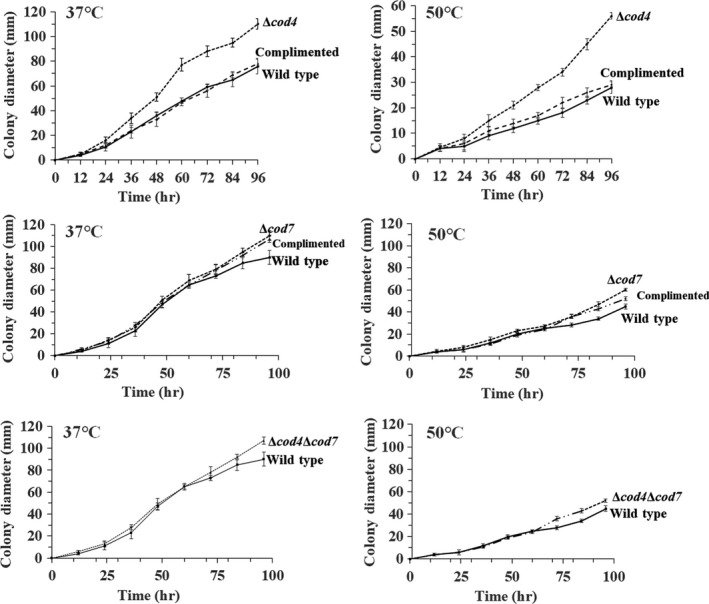
Growth rate of the Δ*cod4*, Δ*cod7*, and Δ*cod4*Δ*cod7* at 37°C and 50°C. A total of 10^6^ fresh conidiospores of each strain were spotted onto the center of a solid CM plate and incubated at 37°C or 50°C, respectively. The diameter of the colony was measured intermittently until the stationary phase, and the mean diameter was used to plot against the growth kinetics. This experiment was carried out in triplicate

To evaluate the physiological function of the *cod4* and *cod7* in *A. fumigatus*, the deletion mutants were constructed by replacing the *cod4* or *cod7* with *pyrG* as described under Section 2, respectively. As a result, the Δ*cod4* and Δ*cod7* were obtained and confirmed by PCR and Southern blotting analysis of*Xba*I‐digested genomic DNA, in which the wild‐type 7.9‐kb*Xba*I fragment was converted into a 5.4‐kb*Xba*I fragment (Figure 5). The double mutant Δ*cod4*Δ*cod7* and revertant strains of the Δ*cod4* and Δ*cod7 *were constructed and confirmed by Southern blot (Figure 4).

The growth rate was determined on solid complete medium at 37°C and 50°C. As shown in Figure 5, the growth rate of the Δ*cod4* mutant was higher than that of the wild type or the revertant strain, while the growth rate of the Δ*cod7* and Δ*cod4*Δ*cod7* was similar to that of the wild type.

**Figure 6:**
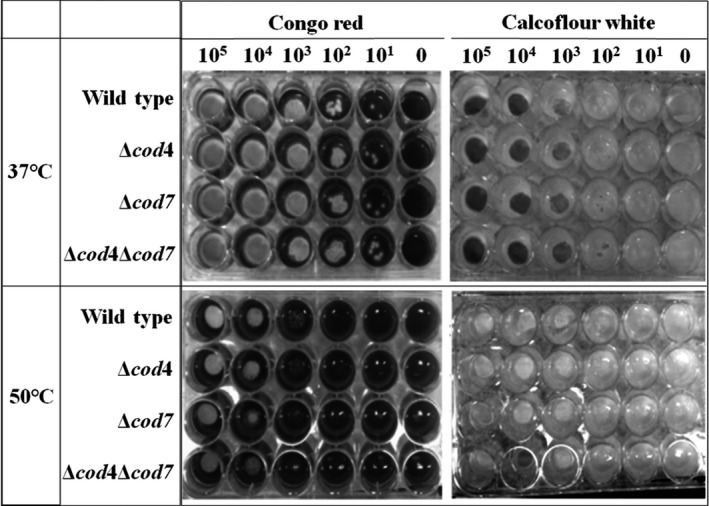
Sensitivity of the Δ*cod4*, Δ*cod7*, and Δ*cod4*Δ*cod7* to chemical compounds. The same number of conidiospores was spotted on CMU in 96‐well plate in the presence of 250 μg calcofluor white ml^−1^ or 250 μg Congo red ml^−1^. After incubation at 37°C or 50°C for 24–48 hr, the plates were taken out and photographed

Considering that CDAs are the enzymes that deacetylate fungal cell wall chitin, we further analyzed the cell wall of the mutants. When the Δ*cod4*, Δ*cod7*, and Δ*cod4*Δ*cod7* mutants were grown on solid complete medium containing calcofluor white or Congo red, all three mutants were similar to the wild type at both 37°C and 50°C (Figure 6). These observations demonstrate that deletion of the *cod4*, *cod7*, or both does not affect the cell wall integrity of *A. fumigatus*.

We further analyzed the cell wall contents of the mutants. As summarized in Table 3, as compared with the wild type, the Δ*cod4* mutant showed decreases in glycoprotein (by 5%) and β‐glucan (by 10%) and increases in α‐glucan (by 8%) and chitin (by 21%). Meanwhile, glycoprotein, α‐glucan, and chitin in the Δ*cod7*decreased by 6%–11%, whereas β‐glucan increased by 6%. The Δ*cod4*Δ*cod7 *showed a similar pattern with the Δ*cod7* mutant but displayed more severe decreases in glycoprotein, α‐glucan, and chitin. Although the cell wall integrity was not affected in the mutants, the cell wall contents were changed in all three mutants. Also Cod4 and Cod7 showed different effects on cell wall contents. Deletion of the *cod4* led to a significant increase in cell wall chitin, while deletion of the *cod4* caused a slight increase in β‐glucan, which suggests their functions might be different.

**Table 3:** Cell wall components of the Δ*cod4*, Δ*cod7*, and Δ*cod4*Δ*cod7* mutants

Strains	Alkali‐soluble		Alkali‐insoluble	
	Glycoprotein (μg)	α‐Glucan (μg)	Chitin (μg)	β‐Glucan (μg)
Wild type	132 ± 3 (100%)	501 ± 27 (100%)	286 ± 21 (100%)	1,182 ± 31 (100%)
Δ*cod4*	125 ± 2 (95%)	543 ± 4 (108%)	345 ± 6 (121%)	1,063 ± 17 (90%)
Δ*cod7*	127 ± 3 (96%)	473 ± 23 (94%)	256 ± 37 (89%)	1,254 ± 25 (106%)
Δ*cod4*Δ*cod7*	115 ± 2 (87%)	443 ± 43 (88%)	235 ± 17 (82%)	1,263 ± 32 (107%)

Note

Three aliquots of 10 mg lyophilized mycelia were used as independent samples for cell wall analysis. The experiments were repeated three times from different biological samples. The values are expressed as micrograms of cell wall component per 10 mg dry mycelia (±*SD*).

Furthermore, both Δ*cod4* and Δ*cod4*Δ*cod7* mutants showed an increased acetylation degree of cell wall chitin, while the Δ*cod7*was similar to the wild type (Table 4). This result is consistent with the observation that Cod7 was inactive on chitin, indicating that Cod4 is an active CDA responsible for the deacetylation of cell wall chitin whereas Cod7 does not act as a CDA enzyme in *A. fumigatus*.

**Table 4:** Acetylation of cell wall chitin in the mutants

Strains	Acetylation (%)
Wild type	58.5 ± 2.3
Δ*cod4*	68.8 ± 2.3
Δ*cod7*	54.3 ± 4.8
Δ*cod4*Δ*cod7*	76.3 ± 3.6

Note

Cell wall chitin was isolated as described under Section 2. Degree of deacetylation of chitosan was determined by the IR spectrophotometry.

### 3.3 Morphogenesis of the mutants

The biological functions of fungal CDAs have been extensively studied in plant pathogenic fungi and yeasts (Cord‐Landwehr et al., 2016; El Gueddari et al., 2002; Zhao et al., 2010). Cbp1, a CDA from the rice blast fungus *M. oryzae*, is confirmed to be critical for appressorium formation, which involves in tip growth and requires accumulation of chitosan at the tips of germ tubes (Kuroki et al., 2017). In *S. cerevisiae*, conversion of chitin to chitosan by either Cda1 or Cda2 is required for formation of the second layer of the spore cell wall, which is important for spores to retain its structural rigidity and resistance to various stresses (Christodoulidou, Briza, Ellinger, & Bouriotis, 1999). CDA from *S. pombe*is also required for proper spore formation (Matsuo et al., 2005), whereas in *C. neoformans*, Cda1, Cda2, and Cda3are confirmed as virulence factorsand responsible for providing cell wall integrity during vegetative growth (Baker et al., 2007; Baker, Specht, & Lodge, 2011; Upadhya et al., 2016).

**Figure 7:**
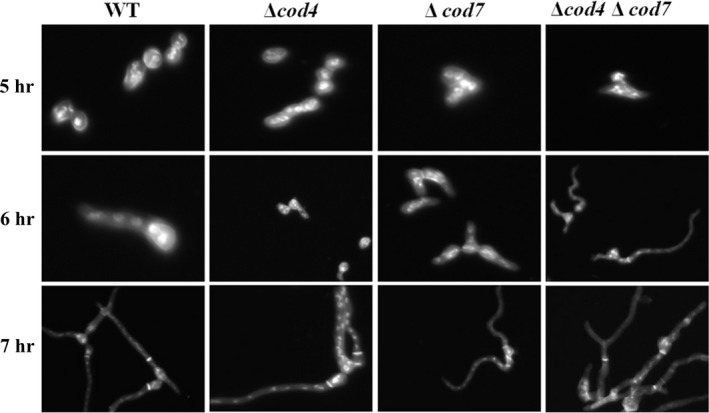
Fluorescent observation of nuclear and cell wall of Δ*cod4*, Δ*cod7*, and Δ*cod4*Δ*cod7* mutants. Ten milliliters of complete liquid medium was inoculated with 10^7^ freshly harvested conidia, poured into a petri dish containing a glass coverslip, and incubated at 37°C for the time indicated in each experiment. At the specified times, the coverslides with adhering germlings were removed and fixed in a fixative solution (4% formaldehyde, 50 mM phosphate buffer, pH 7.0, and 0.2% Triton X‐100) for 30 min. The coverslips with geminated spores were then washed with phosphate‐buffered saline (PBS), incubated for 15 min with DAPI (1 μg/ml; Sigma), washed with PBS three times, then incubated for 5 min with a 10 μg/ml solution of fluorescent brightener 28 (Sigma), and washed again, and the germlings were photographed using a microscope

The developmental process of filamentous fungi is featured with the establishment and maintenance of polarity. The nucleus, meanwhile, undergoes several mitotic divisions, and new nuclei move out into the germ tube. Septation takes place after the third nuclear division by placement of a cross‐wall at the basal end of the germ tube (Harris, Hamer, Sharpless, & Hamer, 1997; Harris, Hofmann, Tedford, & Lee, 1999; Momany & Taylor, 2000). When the *A. fumigatus* wild‐type conidiospores were cultivated in liquid CM media at 37°C, at the early stage of germination, the wild type elongated mostly toward one direction, the second germ tube and the first germ tube showed typically bipolar pattern at an angle of 180 degrees, and the second germ tube and the first septation occurred after four rounds of mitosis (7 hr). The septum formed at the basal area of the first germ tube (Figure 7). In comparison with the wild type, the second germ tube formed earlier in the Δ*cod4*, Δ*cod7*, and double mutant. The second germ tube of all three mutants occurred at a 120° angle only after the second mitosis (5–6 hr), and the third germ tube was found after the third or the fourth nuclear division (6–7 hr). All spores of three mutants germinated at 7 hr and 14%–24% of them had the third germ tube, while all wild‐type spores germinated at 8 hr and only 2% of had the third germ tube (Table 5). These observations suggest that all three mutants germinate earlier than the wild type and display abnormal polarized growth.

**Table 5:** Statistics of germination of the Δ*cod4*, Δ*cod7*, and Δ*cod4*Δ*cod7*

Time (hr)	Wild type number of germ tubes				Δ*cod4* number of germ tubes				Δ*cod7* number of germ tubes				Δ*cod4*Δ*cod7* number of germ tubes			
	0	1	2	≥3	0	1	2	≥3	0	1	2	≥3	0	1	2	≥3
5	58 ± 4	41 ± 3	0	0	38 ± 4	61 ± 3	0	0	27 ± 4	68 ± 3	5 ± 2	0	35 ± 4	63 ± 3	0	0
6	28 ± 2	68 ± 3	4 ± 1	0	8 ± 2	78 ± 3	14 ± 1	0	12 ± 2	58 ± 3	25 ± 1	5 ± 2	11 ± 2	70 ± 3	19 ± 1	0
7	16 ± 3	83 ± 5	5 ± 2	0	0	47 ± 5	39 ± 2	14 ± 2	0	37 ± 5	39 ± 2	24 ± 2	0	44 ± 5	29 ± 2	24 ± 2
8	0	91 ± 4	7 ± 3	2 ± 1	0	51 ± 4	27 ± 3	21 ± 1	0	30 ± 4	37 ± 3	31 ± 1	0	45 ± 4	30 ± 3	25 ± 1

Note

Ten milliliters of liquid CM was inoculated with 10^6^ spores in a petri plate containing sterilized glass coverslips and incubated at 37°C. The coverslips with adhering germinated conidia were taken out, fixed in PFA solution (3.7% paraformaldehyde, 50 mM phosphate buffer, pH 7.0, and 0.1% Triton X‐100), and observed and counted under differential interference contrast microscope.

**Figure 8:**
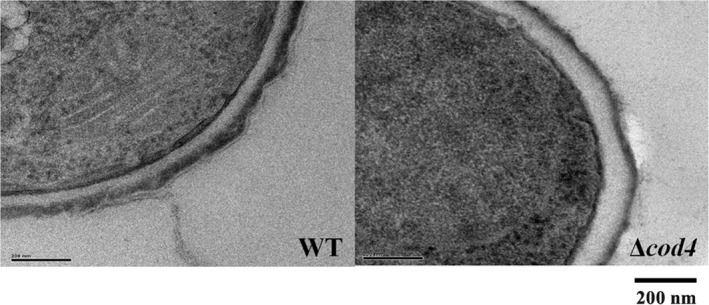
Ultrastructure of conidial cell wall in the Δ*cod4* mutant. The conidia of the wild type and ∆*cod4* mutant produced on solid medium were fixed and examined with H‐600 electron microscope (Hitachi) as described in Section 2

Besides abnormal polar growth, conidiation was also affected in the mutants. At either 37°C or 50°C, the conidiospores produced by the Δ*cod4* and Δ*cod4*Δ*cod7* were dramatically increased, while the conidiospores produced by the Δ*cod7*were similar to those of the wild type (Table 6). Under electron microscope, the conidia of the ∆*cod4*mutant displayed a thickened cell wall (Figure 8). These results indicate that Cod4 is vital to conidiation in *A. fumigatus*. It is likely that the significantly increased conidiospores, as well as thickened conidial cell wall, is a strategy to survive under the stress condition led by deletion of the *cod4*.

**Table 6:** Counting of conidiospores in the mutants

	Number of conidia (×10^8^)			
	Wild type	Δ*cod4*	Δ*cod7*	Δ*cod4*Δ*cod7*
37°C	2.7 ± 0.4	17.6 ± 0.5	3.6 ± 0.3	7.5 ± 0.2
50°C	1.2 ± 0.4	9.1 ± 0.3	1.8 ± 0.3	4.1 ± 0.2

Although Cod7 was inactive on chitin, our results indicate that Cod7 involves in polarity of *A. fumigatus*. A similar result has been observed in *H. pylori*. Although *Hp*PgdA is inactive on peptidoglycan, expression of the *Hp*PgdA encoding gene HP0310 is induced when *H. pylori* is held in contact with macrophages. The HP0310 deletion mutant presents larger acetylation as compared with its wild type and displays an increased susceptibility to lysozyme degradation (Shaik et al., 2011).

**Figure 9:**
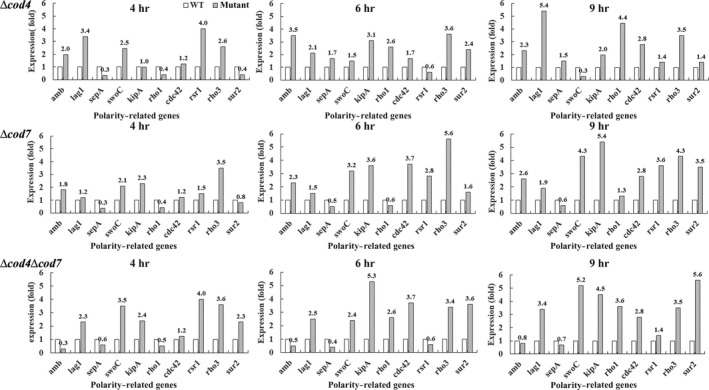
Expression level of polarity‐related gene in the mutants. A total of 10^8^ conidiospores were inoculated into 100 ml of liquid CM medium and incubated at 37°C with shaking (200 rpm). After 4, 6, and 9 hr of cultivation, RNA was extracted and analyzed as described under Section 2. TBP was used as internal standard. The experiments were repeated three times

As the mutants were associated with polarity, we further checked expression of the genes that are involved in the polarized growth of *A. fumigatus* by RT‐PCR. Among the genes tested, *amb*, *rsr1*/*bud1*, *cdc42*, *rho1*, *rho3*, and *sepA*/*bni1* involve in actin rearrangement and are thus required for polarity of fungi, *kipA*involves in regulation of microtublin, and *lag1*, *sur2*, and *swoC *involve in morphology of hyphae. As shown in Figure 9, in the Δ*cod4* mutant, expression levels of all genes except *swoA* were higher than those in the wild type; in the Δ*cod7* mutant, expression levels of all genes except *sepA* were higher than those of the wild type; and in the double mutant, all genes except *amb* and *sepA *were highly expressed. These results confirm that both *cod4* and *cod7 *are required for normal polarized growth of *A. fumigatus*.

It should be pointed out that although both genes contribute to polarity, our results demonstrate that functions of Cod4 and Cod7 are different. Apparently, Cod7 does not act as an enzyme that directly deacetylates cell wall chitin. It is still unknown how Cod7 contributes to polar growth of *A. fumigatus*. We also tested the virulence of both Δ*cod4 *and Δ*cod7* mutants with immunocompromised mouse model; however, we were unable to find any significant change in virulence.

In conclusion, our results suggest that Cod4 is a soluble CDA and involved in polarity and conidiation in *A. fumigatus*. On the other hand, although Cod7 is unable to deacetylate the N‐acetylated polysaccharides recognized by the typical CDAs, it also contributes to polarity of *A. fumigatus*; however, its mechanism needs further investigation.

## CONFLICT OF INTERESTS

None declared.

## AUTHOR CONTRIBUTIONS

CJ conceived, administered, and supervised the project; validated the data with equal contributions from MX and XZ; carried out visualization experiments; and acquired funding. YL developed the methodology, curated the data, and implemented the software; MX and XZ carried out investigation and analyzed the data with support from YL, and wrote the original draft. All authors reviewed and edited the manuscript, and gave the final approval for publication.

## ETHICAL APPROVAL

None required.

## Data Availability

All data generated or analyzed during this study are included in this published article.
